# Using next generation sequencing of alpine plants to improve fecal metabarcoding diet analysis for Dall’s sheep

**DOI:** 10.1186/s13104-021-05590-z

**Published:** 2021-05-07

**Authors:** Kelly E. Williams, Damian M. Menning, Eric J. Wald, Sandra L. Talbot, Kumi L. Rattenbury, Laura R. Prugh

**Affiliations:** 1 School of Environmental and Forest Sciences, University of Washington, Seattle, USA; 2 U.S. Geological Survey, Alaska Science Center, Anchorage, USA; 3 Arctic Network Inventory & Monitoring Division, National Park Service, Fairbanks, USA; 4 Present Address: Department of Evolutionary Anthropology, Duke University, Durham, USA

**Keywords:** Alpine, Arctic, Boreal, Dall’s sheep, Diet, Fecal, Metabarcoding, Chloroplast, Plant

## Abstract

**Objectives:**

Dall’s sheep (*Ovis dalli dalli*) are important herbivores in the mountainous ecosystems of northwestern North America, and recent declines in some populations have sparked concern. Our aim was to improve capabilities for fecal metabarcoding diet analysis of Dall’s sheep and other herbivores by contributing new sequence data for arctic and alpine plants. This expanded reference library will provide critical reference sequence data that will facilitate metabarcoding diet analysis of Dall’s sheep and thus improve understanding of plant-animal interactions in a region undergoing rapid climate change.

**Data description:**

We provide sequences for the chloroplast *rbcL* gene of 16 arctic-alpine vascular plant species that are known to comprise the diet of Dall’s sheep. These sequences contribute to a growing reference library that can be used in diet studies of arctic herbivores.

**Supplementary Information:**

The online version contains supplementary material available at 10.1186/s13104-021-05590-z.

## Objective

Dall’s sheep (*Ovis dalli dalli*) are endemic to alpine ecosystems of northwestern North America, and their populations have been declining in recent decades [1–4]. Climate change may be altering alpine plant communities and contributing to these declines. Dall’s sheep have a generalist plant diet; they were observed eating 110 different plant species in the Yukon Territory, Canada through traditional observational methods [5]. However, the diet of Dall’s sheep remains relatively poorly characterized and represents a gap in understanding how climate change is affecting plant-animal interactions in alpine ecosystems.

The level of taxonomic resolution of items consumed in a diet study greatly affects ecological analysis [6]. DNA based tools can infer diet composition with higher resolution and reduces cost, time, and effort compared to observational, morphological, and microhistological methods [7, 8]. Specifically, DNA metabarcoding uses universal primers for multispecies identification to mass-amplify DNA barcodes using PCR that are then read using next generation sequencing and assigned to the appropriate taxon [9]. DNA barcoding includes a reference database of potential diet components, providing the capability to identify diet items to a desirable taxonomic resolution, ensuring that all components will be detected and assigned [10]. Next generation sequencing of DNA from fecal samples has been successfully used to characterize diets of a variety of species, including ungulates [11, 12]. However, metabarcoding has not yet been used to assess the diet of Dall’s sheep. Lack of sequence data for some arctic/alpine plants known to be grazed upon by Dall’s sheep currently limits the development and application of metabarcoding for alpine herbivore diet studies.

To improve capabilities for diet analysis of Dall’s sheep and other arctic herbivores, we used a python script [13] to identify gaps in archived nucleotide sequence data for species known to comprise the diet of Dall’s Sheep, then obtained specimens of 16 species of arctic/alpine vascular plants for which sequence information was missing or underrepresented in publicly archived databases. We then sequenced the *rbcL* gene of the plant chloroplast genome, which is one of the most commonly used barcoding regions for plants [9, 14].

## Data description

Plant specimens were obtained from herbarium specimens collected from the various arctic or alpine sites across mainland Alaska (Additional file 1). Plant tissue was extracted at the U. S. Geological Survey Alaska Science Center, employing a CTAB-PVP protocol modified from Stewart and Via [15] as reported by Muñiz-Salazar et al. [16]. Extracts were quantified and shipped to the School of Environmental and Forest Sciences Genetics Lab at the University of Washington for PCR amplification and NexteraXT library preparation for sequencing. The *rbcL* gene region of each specimen was amplified via a two-step PCR protocol [17] with a primary amplification with tailed primers (rbcLaf + adaptor, rbcLr506 + adaptor) followed by a second round of amplification to anneal NexteraXT indices. Amplicons were quantified using a Qubit 4 Fluorometer (ThermoFisher) and diluted with dH2O to the recommended starting concentration for library preparation, 0.2 ng/μL (Illumina). Tagmentation, library amplification, and clean-up steps were completed according to the NexteraXT library preparation protocol (Illumina) with a variation of using New England Biolabs AMPure XP beads for cleanup instead of Agentcourt AMPure beads. The libraries were normalized and pooled prior to sequencing on an Illumina Miseq platform. Samples were paired-end sequenced in a 2 × 300 bp format .

Illumina sequence reads were processed in Geneious Prime 2020.2.4. Forward and reverse read files (fastq) were paired upon import, then quality trimmed with BBDuk trimmer (minimum quality 20, minimum overlap 20, minimum length 20). Sequences were normalized, then aligned and assembled using the de novo assembly tool (Geneious Prime). Assembled contigs were uploaded and annotated using BankIt, then submitted to GenBank [18] (Table 1).

**Table 1:** Overview of data files for arctic plant *rbcL* sequencing

Label	Name of data file/data set	File types	Data repository and identifier
*Data file 1*	Elymus borealis rbcl contig	*.gb	https://identifiers.org/ncbi/insdc:MW538513 [19]
*Data file 2*	Gentiana propinqua rbcl contig	*.gb	https://identifiers.org/ncbi/insdc:MW538515 [20]
*Data file 3*	Juncus mertensianus rbcl contig	*.gb	https://identifiers.org/ncbi/insdc:MW548523 [21]
*Data file 4*	Luzula arctica rbcl contig	*.gb	https://identifiers.org/ncbi/insdc:MW548524 [22]
*Data file 5*	Ranunculus kamchaticus rbcl contig	*.gb	https://identifiers.org/ncbi/insdc:MW548525 [23]
*Data file 6*	Oxytropsis scammaniana rbcl contig	*.gb	https://identifiers.org/ncbi/insdc:MW548526 [24]
*Data file 7*	Packera ogotorukensis rbcl contig	*.gb	https://identifiers.org/ncbi/insdc:MW548527 [25]
*Data file 8*	Penstemon gormanii rbcl contig	*.gb	https://identifiers.org/ncbi/insdc:MW548528 [26]
*Data file 9*	Saxifraga caespitosa rbcl contig	*.gb	https://identifiers.org/ncbi/insdc:MW548529 [27]
*Data file 10*	Silene tayloriae rbcl contig	*.gb	https://identifiers.org/ncbi/insdc:MW548530 [28]
*Data file 11*	Smelowskia integrifolia rbcl contig	*.gb	https://identifiers.org/ncbi/insdc:MW548531 [29]
*Data file 12*	Stellaria alaskana rbcl contig	*.gb	https://identifiers.org/ncbi/insdc:MW548532 [30]
*Data file 13*	Taraxacum lyratum rbcl contig	*.gb	https://identifiers.org/ncbi/insdc:MW548533 [31]
*Data file 14*	Anemone lithophilia rbcl contig	*.gb	https://identifiers.org/ncbi/insdc:MW526257 [32]
*Data file 15*	Carex pyrenaica rbcl contig	*.gb	https://identifiers.org/ncbi/insdc:MW538514 [33]
*Data file 16*	Elymus latiglumis rbcl contig	*.gb	https://identifiers.org/ncbi/insdc:MW537582 [34]

## Limitations

The following are limitations for these data files:

1. We sequenced one DNA extraction from each plant species.
2. The sequencing project was funded through a grant to train new users on Illumina Nextera sequencing.

## Supplementary Information

**Additional file 1.** Table of information about the plant specimens used for rbcl sequencing.

## Data Availability

Please see Table 1 and references [19–34] for details and links to the data. Please see Additional file 1 for a table of information about the plant specimens used for sequencing.

## Abbreviations

rbcL: Large subunit of ribulose 1, 5 bisphosphate carboxylase/oxygenase (RUBISCO or RuBPCase)
CTAB-PVP: DNA extraction method using cetyltrimethylammonium bromide as a detergent-based extraction buffer and polyvinylpyrrolidone, which is added to remove phenolic compounds from plant DNA extracts [15, 16]
PCR: Polymerase chain reaction
NexteraXT: NexteraXT DNA library preparation kit enables sequencing of small genomes, PCR amplicons, and plasmids (Illumina)
Miseq: Illumina Miseq Next Generation Sequencer is an integrated instrument that performs clonal amplification, genomic DNA sequencing, and data analysis with base calling, alignment, variant calling, and reporting in a single run (Illumina)
